
JOURNAL INFORMATION
==============================
NLM Title Abbreviation: ScientificWorldJournal
Journal ID: ScientificWorldJournal
NLM Journal ID: 101131163
Journal ID: TSWJ

The Scientific World Journal

ISSN: 2356-6140
EISSN: 1537-744X
Publisher: Wiley

ARTICLE INFORMATION
==============================
PMCID: PMC5763785
PMID: 20563527
DOI: 10.1100/tsw.2010.109
Article ID: 147304
Article version: 1
Subjects: Letter to the Editor

Progressive Familial Intrahepatic Cholestasis (PFIC) Type 3 in Two Sicilian Siblings of Nonconsanguineous Parents

Crupi Isodiana *
Pediatrician, San Piero Patti, Messina, Italy
*Isodiana Crupi: crupi.isodi@libero.itAcademic Editor: Raj K. Goyal

Publication date: 2010
Electronic publication date: 2010 Jun 14
Volume: 10
First page: 1065
Last page: 1066
Received 2010 Jan 24; Revised 2010 Apr 19; Accepted 2010 May 11
Copyright: Copyright © 2010 Isodiana Crupi.
Copyright year: 2010
License: This is an open access article distributed under the Creative Commons Attribution License, which permits unrestricted use, distribution, and reproduction in any medium, provided the original work is properly cited.
License URL: https://creativecommons.org/licenses/by/3.0/

Keywords: cholestasis, biliary cirrhosis, autosomal-recessive disorders

==============================
Letter to the Editor
*TheScientificWorldJOURNAL* (2010) 10, 1065–1066
ISSN 1537-744X; DOI 10.1100/tsw.2010.109

TheScientificWorldJOURNAL
www.thescientificworld.com

### Progressive Familial Intrahepatic Cholestasis (PFIC) Type 3 in Two Sicilian Siblings of Nonconsanguineous Parents

Isodiana Crupi
*Pediatrician, San Piero Patti, Messina, Italy*

**E-mail**: crupi.isodi@libero.it

*Received January 24, 2010; Revised April 19, 2010; Accepted May 11, 2010; Published June 14, 2010*

**KEYWORDS**: cholestasis, biliary cirrhosis, autosomal-recessive disorders

Progressive familial intrahepatic cholestasis (PFIC) is a group of three types of autosomal-recessive disorders, characterized by cholestasis and biliary cirrhosis due to defects in specific transport proteins involved in bile formation[1,2,3]. Clinical presentation usually occurs first in childhood, with progressive cholestasis that leads to failure to thrive, hepatic failure, and liver transplantation. PFIC type 3 is caused by a variety of mutations in ABCB4 (ATP-binding cassette, subfamily B, member 4), the gene encoding multidrug resistance protein 3 (MDR3), which codes for flippase, an enzyme responsible for the transport of phospholipid molecules between cell membranes. Initial treatment with ursodeoxycholic acid, cholestyramine, rifampin, and fat-soluble vitamins is supportive, while patients must have a liver transplant when liver dysfunction is significant[4,5].

Two brothers, affected by PFIC type 3, were referred to the pediatrician. The first was diagnosed at the age of 4 years. He presented no signs or symptoms until, at the age of 1 year, due to a swollen abdomen and failure to thrive, he was prescribed laboratory tests (antigliadin antibodies, antiendomysial antibodies) and a sweat test that all gave normal results. Later, possibly due to an allergic reaction with urticaria, he was given antihistaminic therapy, with no relief. This led to referral to an allergologist, who prescribed a series of blood exams that showed AST 165 UI/l (normal range: 15–55), ALT 180 UI/l (5–45), and γ-GT 80 UI/l (5–32). The child was then admitted to a pediatric gastroenterology center for further examination. After a series of exams to exclude other causes of liver disease, the child was referred to a pediatric hepatology center for a liver biopsy that resulted in a diagnosis of PFIC type 3. Genomic DNA analysis showed homozygosity for mutations in the human MDR3 gene with heterozygosity of the nonconsanguineous parents.

In the second brother, the disorder was diagnosed at birth, the only sign being a prolonged jaundice. Blood exams showed elevated serum liver enzymes and increased γ-GT activity (three times the normal values). Genomic DNA analysis showed homozygosity for the same mutations of the sibling in the human MDR3 gene. Both brothers, with nonconsanguineous parents, are in good clinical condition at 11 years and 1 year of age, respectively. They are under medical treatment with cholestyramine ursodeoxycholic acid, and fat-soluble vitamins.

De Vree et al.[3] reported two cases of PFIC type 3: the first was a Turkish boy of healthy consanguineous parents; the second, a North African boy of first-cousin parents. To our knowledge, the

©2010 with author.
Published by TheScientificWorld; www.thescientificworld.com presence of mutations in the human MDR3 gene has been described so far only in siblings of consanguineous parents.

### ACKNOWLEDGMENTS

The author wishes to thank Dr. Lorenzo Curtò for his contribution to the management of the paper.

### REFERENCES

1. Jansen, P.L. and Muller, M.M. (1998) Progressive familial intrahepatic cholestasis types 1, 2 and 3. *Gut* **42**, 766–767.
2. A-Kader, H.H. and Balistreri, W.F. (2004) Cholestasis. In *Nelson Textbook of Pediatrics*. 17th ed. Behrman, R.E., Kliegman, R.M., and Jenson, H.B., Eds. WB Saunders, Philadelphia. pp. 1314–1319.
3. De Vree, J.M., Jacquemin, E., Sturm, E., et al. (1998) Mutations in the MDR3 gene cause progressive familial intrahepatic cholestasis. *Proc. Natl. Acad. Sci. U. S. A.* **95**, 282–287.
4. Davit-Spraul, A., Gonzales, E., Baussan, C., and Jacquemin, E. (2009) Progressive familial intrahepatic cholestasis. *Orphanet J. Rare Dis.* **4**, 1.
5. Oude Elferink, R.P. and Paulusma, C.C. (2007) Function and pathophysiological importance of ABCB4 (MDR3 P-glycoprotein). *Pflugers Arch.* **453,** 601–610.

**This article should be cited as follows:**

Crupi, I. (2010) Progressive familial intrahepatic cholestasis (PFIC) type 3 in two Sicilian siblings of nonconsanguineous parents. *TheScientificWorldJOURNAL* **10**, 1065–1066. DOI 10.1100/tsw.2010.109.
